# Draft genome of *Talaromyces* sp. UPCC 4333, from *Senna alata* flowers, displayed a remarkable diversity of potentially novel biosynthetic gene clusters

**DOI:** 10.1128/mra.01055-24

**Published:** 2024-12-17

**Authors:** Rafael B. Eugenio, Paul Christian T. Gloria, Mary Ann Cielo V. Relucio-San Diego

**Affiliations:** 1 Natural Sciences Research Institute, University of the Philippines 54727 , Quezon, Philippines; University of California Riverside, Riverside, California, USA

**Keywords:** *Talaromyces*, whole genome, draft genome, biosynthetic gene clusters

## Abstract

Here, we report the draft genome of *Talaromyces* sp. UPCC 4333, isolated from *Senna alata* flowers, with a size of 31.3 Mb and 92.9% BUSCO completeness. The genome contains at least 39 secondary metabolite biosynthetic gene clusters which merit further investigation in the future.

## ANNOUNCEMENT

The genus *Talaromyces* is known to be a prolific producer of secondary metabolites. Some species reportedly have anticancer, antibacterial, antifungal, antiproliferative, biocontrol, plant-growth promotion, enzymes, colorants, and antioxidant compounds (1–9). Most activities were discovered using classical screening methods via bioassays depending on target activities, but the utilization of genomics demonstrates much greater potential than traditional means (10). For this reason, we sought to sequence the genome of *Talaromyces* sp. UPCC 4333.

*Talaromyces* sp. UPCC 4333 was isolated from the flowers of *Senna alata* collected from the grounds of the University of the Philippines, Diliman, Philippines (14.6449 N, 121.0701 E) by macerating the plant tissue, serially diluting with 0.1% peptone water, and plating on Czapek Dox Agar, which served as its maintenance medium. The isolate was grown in the Czapek Dox Broth at room temperature for 7 days. Afterwards, approximately 500 mg of mycelia were collected for DNA extraction using DNeasy PowerSoil Pro Kit (QIAGEN, USA) for its proven ability to extract high DNA yields in our laboratory. Extracted DNA was sent to Macrogen, South Korea, for sequencing. Library was prepared using TruSeq Nano DNA Kit and sequencing commenced in an Illumina NovaSeq 6000 platform, 151 pair-ended settings, with at least 5 Gb throughput.

All bioinformatics tools were used under default parameters unless specified otherwise. Raw reads were adapter- and quality-trimmed to Phred30 using Trimmomatic v.036 (11). Q30-trimmed reads were assembled in SPAdes v3.15.5 (12). Assembly quality was evaluated using QUAST v5.2.0 (13) and BUSCO v.5.7.1 in mode *euk_genome_met,* and the gene predictor used was *metaeuk* (14). Masked assembly was generated using RepeatMasker v4.1.5 (15). Lastly, annotations were generated through Braker3 v.3.0.6 (16). AntiSMASH fungal version (fungiSMASH) v7.1.0 (17) was utilized to predict secondary metabolite biosynthetic gene clusters (smBGCs) in the genome.

Sequencing yielded 41,872,608 reads (6.3G bases) with 97.3% and 92.6% at Q20 and Q30, respectively. The assembly process produced a 31.3 Mb genome with a GC content of 43.5%. The full assembly contains 118 contigs, an N_50_ of 1,003,615 bp, an L_50_ of 12, and coverage of 52×. The largest contig’s size is at 2,384,676 bp. BUSCO analysis indicated 92.9% completeness where out of 4,191 BUSCO groups searched, 3,896 are complete of which 9 are duplicated while 3,887 are single-copy. Thirty-six BUSCO groups were fragmented. A total of 9,945 genes were predicted using Braker3. Thirty-nine smBGCs were predicted by fungiSMASH where fungal RiPP-like (10) and T1PKSs (10) are most abundant followed by NRPSs (seven) and NRPS-like (six) regions. Of the 39 regions, 5 had 100% similarity to its closest known cluster hits, while the rest had less than 40% similarity which highlights the extreme diversity and novelty of smBGCs within the *Talaromyces* sp. UPCC 4333 genome.

## Data Availability

This genome project has been registered at NCBI under accession number PRJNA1109622. Raw reads were deposited at the NCBI Sequence Read Archive (SRA) under the accession number SRR29341196. The genome sequence has been deposited at GenBank under the accession number JBEPII000000000 with the assembly accessioned under ASM4066993v1. Lastly, annotations have been uploaded in Zenodo (18).
